# Integration of passive sensing technology to enhance delivery of psychological interventions for mothers with depression: the StandStrong study

**DOI:** 10.1038/s41598-024-63232-3

**Published:** 2024-06-12

**Authors:** Alastair van Heerden, Anubhuti Poudyal, Ashley Hagaman, Sujen Man Maharjan, Prabin Byanjankar, Dörte Bemme, Ada Thapa, Brandon A. Kohrt

**Affiliations:** 1 Center for Community Based Research, Human Sciences Research Council, Pietermaritzburg, South Africa; 2 South African Medical Research Council/Wits Developmental Pathways for Health Research Unit, Department of Paediatrics, Faculty of Health Sciences, University of the Witwatersrand, Johannesburg, South Africa; 3 Department of Sociomedical Sciences, Columbia Mailman School of Public Health, New York, NY USA; 4 Department of Psychiatry and Behavioral Sciences, Center for Global Mental Health Equity, George Washington School of Medicine and Health Sciences, Washington, DC USA; 5 Department of Social and Behavioral Sciences, Yale School of Public Health, Yale University, New Haven, CT USA; 6 Center for Methods in Implementation and Prevention Science, Yale University, New Haven, CT USA; 7 Transcultural Psychosocial Organization Nepal, Kathmandu, Nepal; 8 Department for Global Health and Social Medicine, Kings College London, London, UK; 9 Division of Global Health Equity, Brigham and Women’s Hospital Boston, Boston, MA USA

**Keywords:** Health services, Psychology

## Abstract

Psychological interventions delivered by non-specialist providers have shown mixed results for treating maternal depression. mHealth solutions hold the possibility for unobtrusive behavioural data collection to identify challenges and reinforce change in psychological interventions. We conducted a proof-of-concept study using passive sensing integrated into a depression intervention delivered by non-specialists to twenty-four adolescents and young mothers (30% 15–17 years old; 70% 18–25 years old) with infants (< 12 months old) in rural Nepal. All mothers showed a reduction in depression symptoms as measured with the Beck Depression Inventory. There were trends toward increased movement away from the house (greater distance measured through GPS data) and more time spent away from the infant (less time in proximity measured with the Bluetooth beacon) as the depression symptoms improved. There was considerable heterogeneity in these changes and other passively collected data (speech, physical activity) throughout the intervention. This proof-of-concept demonstrated that passive sensing can be feasibly used in low-resource settings and can personalize psychological interventions. Care must be taken when implementing such an approach to ensure confidentiality, data protection, and meaningful interpretation of data to enhance psychological interventions.

## Introduction

Depression is one of the most prevalent psychiatric disorders in the postpartum period with an estimated 3–32% prevalence among women in low- and middle-income countries (LMICs)^1^. In LMICs, lack of treatment for postpartum depression (PPD) is associated with a range of negative mental and physical health outcomes and considerable distress and disability for young mothers and their children, including low responsiveness towards the infant and poor infant cognitive development PPD is recognised as a significant risk factor for the child of a depressed mother to develop depression themselves later in life. With poor maternal and child health outcomes, the need to develop effective strategies to reduce PPD is critical.

Although there are several effective interventions to manage depression, one of the greatest challenges in the field of mental health is the lack of skilled mental health professionals to deliver these interventions to those in need^2^. There has been development and testing of interventions for PPD delivered by paraprofessionals in LMICs^3^. However, non-specialist interventions have shown mixed results, and limited long-term benefits^4^. Many of the currently used interventions delivered by non-specialists, such as lay counsellors, are rooted in the theory of behavioural activation (BA)^3^. BA helps individuals identify their most important goals, clarify why these goals are important and then identify behaviours that will help them move towards achieving their goals^5^. BA is a highly flexible process, and counsellors can adapt it to individuals’ circumstances. By focusing on goals and behaviour, BA is a potentially useful intervention for lay counsellors to deliver to individuals with PPD. The challenge is to identify the different circumstances that individuals face and how these circumstances may impact their mental health. Passive sensing technologies in mobile phones and non-invasive wearable devices have the potential to supplement BA interventions by collecting data that can be transformed by machine learning to enhance the delivery of psychological interventions by monitoring behavioural patterns and reinforcing behavioural change^6^.

Digital phenotypes are the biographical data profiles of individuals generated from passively collected personal data. They provide the person-context pairs that define the individual’s moment-by-moment situated experiences in their physical, digital and social environments^7^. Analysis of passively collected data, such as mobile phone usage, can provide insight into the challenge’s individuals face in their everyday lives and the effects of those challenges on their mental health. This may be used to tailor interventions to the individual’s needs and circumstances^8,9^.

To illustrate, passive data collection through wearable devices has been utilized to monitor sleep patterns, revealing correlations between sleep disturbances and mood disorders^10,11^. This approach enables the tailoring of interventions focused on improving sleep hygiene, which is crucial in treating mood disorders. Similarly, monitoring physical activity through smartphones and wearable devices has been instrumental in identifying the relationship between decreased physical activity and depressive symptoms^12^. As one of the most important features in models designed to estimate depressive symptoms^12^, activity data could facilitate the development of interventions that encourage and monitor physical activity, addressing a key aspect of depression management. Furthermore, passive data collection has proven effective in monitoring social rhythms in individuals with schizophrenia and bipolar disorder, providing insights into changes in social behaviour. By modelling this metric from digital footprints, interventions can be personalized to help stabilize daily routines without the need for self-reporting^13,14^. Lastly, voice analysis through passive data collection offers a novel approach to detecting affective disorders, enabling tailored interventions that focus on emotion regulation techniques and provided timely support when signs of emotional distress are identified^15^.

Mobile phone-based passive sensing enables measurement of human mobility through the global positioning system (GPS), physical activity through accelerometers, exposure to environmental sounds including human speech through processed audio recordings, and proximity to children and others using Bluetooth technology. Further, physiological data (e.g., heart rate, sleep patterns) can be collected from wearables such as smartwatches. These data can all be collected relatively unobtrusively from the individual and used to create a personal data profile that represents the individual’s behavioural characteristics^16,17^. These data are available through edge devices such as smartphones and smartwatches and uploaded to the cloud where they are processed, combined and analysed using machine learning approaches such as multilinear principal component analysis and convolutional neural networks to provide information for paraprofessionals delivering psychological interventions. In this study, we aimed to integrate a passive sensing platform that collects audio, GPS location, mother–child proximity, and physical activity into a psychological intervention delivered by paraprofessional counsellors in rural Nepal to test for proof of concept^6^.

## Methods

### Study design and participants

The proof-of-concept study followed formative development work^18–20^. This paper reports on the final single-arm intervention where the StandStrong counsellor app^19^ was provided to lay counsellors offering a 5-session BA intervention, entitled the Healthy Activity Program (HAP)^21^, to systematically visualize the passive sensing data of depressed adolescent and young mothers during care. The passive sensing data were designed to be used to provide tailored counselling sessions to the mothers^6^. All research was conducted in consultation with a community advisory board made up of female community health volunteers and auxiliary nurse midwives from seven health facilities.

### Study setting

Nepal is classified as a lower-middle-income country and among the poorest in South Asia^22^. Mental health services are not widely available in this context, though Nepal does lead compared to similar LMIC counterparts with recent mental health policies and primary mental health service implementation. In larger cities, some government and private hospitals offer services^23^. In Chitwan, a district in the Bagmati Province, a southern region of Nepal, specialist mental health services are provided by two psychiatrists and a psychiatric ward in the district hospital. These resources see Chitwan having more capacity than most other areas in Nepal. This is in part because, for the past decade, Chitwan has been the Nepal implementation district for the Program for Improving Mental Health Care (PRIME). The psychological treatment for postpartum depression that was offered in this study emerged from intervention trials as part of PRIME^24^.

### Intervention and mHealth platform

#### The healthy activity programme (HAP)

**Figure 1:**
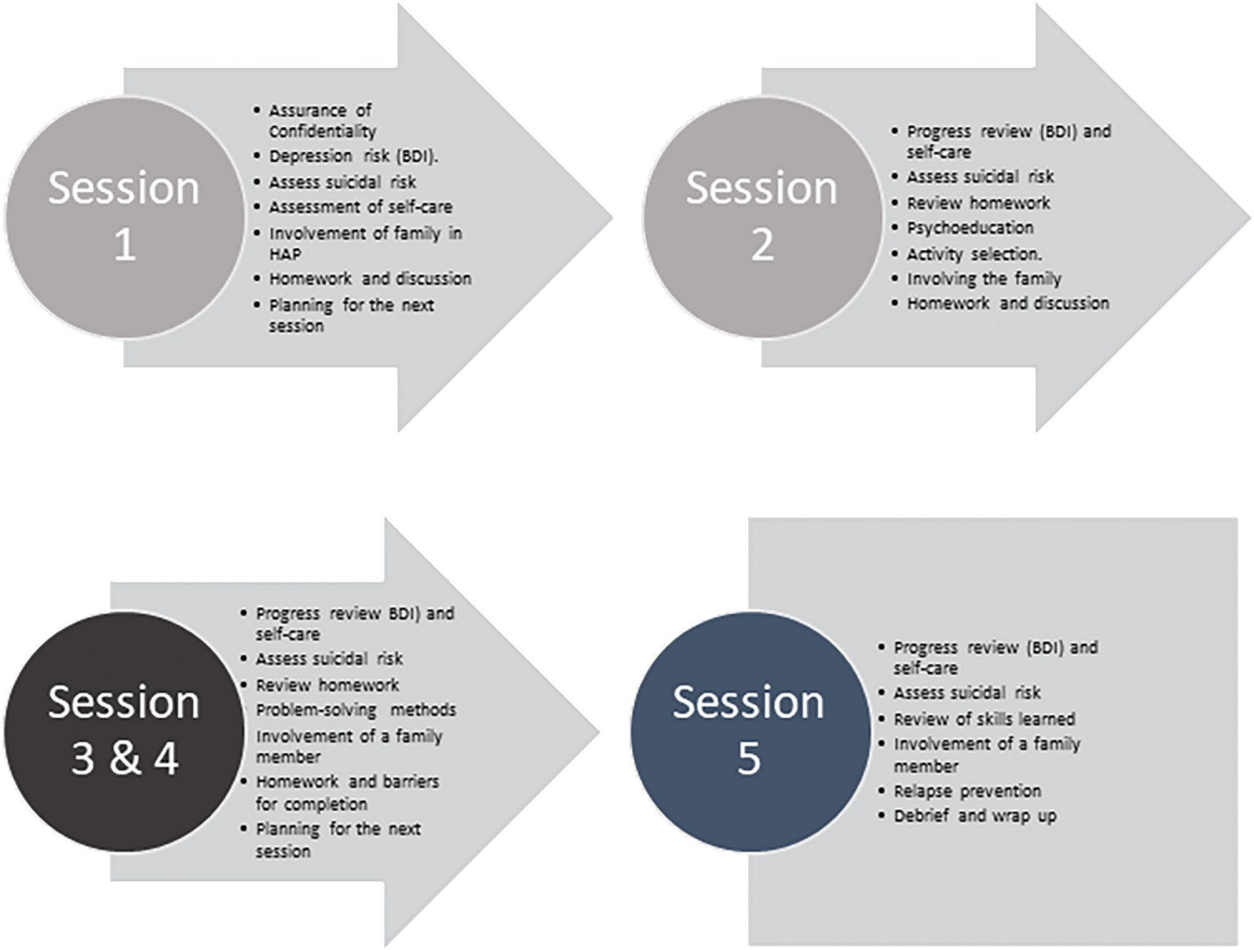
Schematic overview of the content covered in each of the five Healthy Activity Programme sessions as implemented in Nepal.

HAP consists of approximately 5–8 sessions divided into three phases. A comprehensive overview of the HAP intervention development has been previously published^21^. In summary (Fig. 1), Phase I (assessment and psychoeducation) is completed over the course of two sessions. Session 1 introduces Initial assessments for psychosocial well-being and self-care. Session 2 follows a week later with Psychoeducation based on HAP and self-care. Phase II also contains two sessions both focusing on problem solving. Finally, in Phase III, a single session is spent focusing on relapse prevention & wrap-up.

#### Electronic behaviour monitor app

Passive sensing data was collected using a low-cost Android smartphone and a Bluetooth Low Energy beacon (Radius Networks) attached in a pouch to the child’s clothing. The Electronic Behaviour Monitor (EBM) v2.0 was installed on the smartphone and configured to run every 15 min between 4:00 AM and 9:59 PM daily. The EBM app passively collected data for 30 s every time it was triggered for 18 h a day. Data collected are short audio recordings (.wav) from the mobile phone mic, GPS location using the Android LocationManager (lat, lon, accuracy), mother–child proximity established by scanning from the mobile phone running the EBM for Bluetooth advertising packets (id, RSSI) from a Bluetooth beacon (RadBeacon, Radius Networks), and physical activity (walking, running, tilting, still, driving, unknown) using the Activity Recognition API provided by Android. As network connectivity was a concern, the EBM was designed as an offline-first app with all data being saved to a folder on the smartphones SD card. A research assistant visited the home once a week to download the data from the SD card and then uploaded it to the study cloud database on return to the office^19^. More information about the EBM platform and findings relating to the acceptability and feasibility of the approach are available^20,25^.

#### StandStrong platform

The Sensing Technologies for Maternal Depression Treatment in Low Resource Settings (StandStrong) Provider Platform was designed to surface behavioural insights about how HAP was affecting participants behaviour^19^. The platform gives providers easy access to review the passive data collected by the EBM app and thereby provides personalized HAP sessions to mothers. The app’s home screen was a feed of data cards designed to visualize the passively collected data. Data cards included visualisations for proximity, activity, GPS movement and proximity / daily routine. Finally, when goals (similar to setting a 10,000-step walking goal) were achieved, the provider was notified and they were able to directly message the participant from within the StandStrong App to the participants Viber, a popular messaging app in Nepal.

#### Assessment tools

The Patient Health Questionnaire (PHQ-9) was administered as part of the screening assessment to confirm eligibility. The PHQ-9 is a widely used tool for assessing depression^25,26^. It contains 9 items rated on a 0 to 3 frequency scale. In Nepal, a cut-off score of 9 or more was selected for this study based on validation findings from a primary care population in Chitwan; this cut-off yields a 94% sensitivity and 69% specificity^27^. The Beck Depression Inventory (BDI) is a 21-item scale rated on 0 to 3 severity scale^28^. The BDI has been validated for use in Nepal with an established cut-off score of 10 for moderate depression (sensitivity = 0.73, specificity = 0.91)^29^. The BDI was selected as the primary outcome for this study because it is routinely used in clinical practice in Nepal as a measure of symptom improvement and the diversity of items allowed for more comprehensive symptom monitoring compared to the PHQ-9 (i.e., 21 symptoms in the BDI, including behavioural symptoms, compared to 9 symptoms in the PHQ). The World Health Organization Disability Assessment Schedule 2.0 (WHODAS 2.0) was used to assess impairment in functioning due to health problems in the past 30 days^30^. It is a 12-item scale and provides with 5-point response score from 1 ‘none’ to 5 ‘Extreme/cannot do’^31^. The WHODAS has been adapted^32^, and used widely been used in Nepal^33^. These locally adapted and validated scales were supplemented with qualitative data collected through in-depth interviews with participating mothers and providers.

### Ethics declaration

This study was approved by the Nepal Health Research Council (#327/2018) and George Washington University's Institutional Review Board (#051,845). All methods were performed in accordance with the relevant guidelines and regulations of both IRBs and countries. Informed consent was obtained from all participants.

### Procedures

Female research assistants recruited adolescent mothers from postnatal clinics and immunization camps in Chitwan, Nepal. After informed consent, the PHQ-9 was administered as a screening tool. In addition to requiring a PHQ-9 score higher than or equal to 9, eligibility criteria included (a) mothers between the ages of 15–25 years, and (b) infants younger than 12 months of age. Once a participant was screened for depression and informed consent completed, a study team member visited her home for family informed consent. For participants under the age of 18 years, we asked for a written parental permission form and assent form. Once enrolled, the mothers received passive sensing technology (smartphone with EBM app installed and Bluetooth beacon on the baby) and enrolled in a 5-week HAP programme. During the sessions, the counsellor used the passive sensing data to tailor the therapy and incorporated the data visualization to share the mother’s behavioural changes. The counsellor used the data with the participants in the sessions to keep track of the behavioural changes, for example, they used it for mood monitoring associated with their daily activities. The participant would maintain a log of their daily life and then also rate their mood on how they felt at that time. The discussion of these data was then supplemented by the counsellor using the passive data looking at if they left the house, how far they walked, and if they spent time with their child. They also jointly reviewed the GPS map which showed a heatmap of hot spots that were frequently visited outside of their homes which introduced interesting discussions about physical activity and social support. The counsellor used this passive data to enrich and probe further into the client’s self-reports.

For the passive data collection, audio was assessed for the presence or absence of speech using the Pyannote Voice Activity Detection (VAD) model^34^. The activity was classified into 7 categories using the Activity Recognition API by Google on the Android platform. Proximity information was collected by recording RSSI values, a measure of signal strength, between the beacon worn by the child and the smartphone carried by the mother. These data were recoded into a binary categorical variable (presences/absence). Finally, GPS data was processed using the scikit-mobility Python package^35^.

### Analysis

The primary outcome, to demonstrate the effectiveness of the tech-enhanced HAP intervention, was the BDI. We intended to recruit 26 depressed mothers to reliably determine these outcomes. Sample characteristics at baseline including BDI, WHODAS, mothers' education and occupation, child’s age, and primary caregiver were all summarised with descriptive statistics. Clinical change and survival analyses for the primary outcome (BDI) were performed using SPSS 24. Passive data were aggregated by week and changes over time were calculated using pandas in Python 3.8. Correlation matrices and change statistics were calculated for the primary clinical outcome (BDI) along with changes in passive data using Spearman rho due to the non-normality of data. A time to remission approach was used for the survival analysis. For each participant, weekly BDI scores were recorded, with the 'event' occurring at the first instance of a BDI score falling below the established cutoff point. For participants who did not achieve a score below the cutoff during the study, their data were right-censored at the last observation point. Qualitative data were inductively coded by two independent coders to produce a set of emerging themes. A more complete analysis of the qualitative data is planned for a future paper. Thematic codes relating to the usefulness of the passive data in the delivery of care were extracted for this paper.

## Results

A total of 782 mothers were screened at health posts during infant immunization camps. Among the mothers screened, 320 were 15–25 years old, and had an infant under 12 months (Table 1). There were 26 categorized as depressed (PHQ-9 ≥ 9). Two dropped out before data collection began and were not included in the analysis. Of the remaining 24 mothers included in the analysis, the mean age of the mother was 21 (SD 2.06) with 17 (70.83%) having a secondary level of education. Mothers had been married on average 4.46 years and had between one and three children, with the oldest child averaging 6.06 years old. Only 2 (8.33%) of the mothers had husbands who were unemployed, with 3 (12.50%) working abroad and spending long periods away from home. Fifty percent of the households had 4 occupants with a maximum household occupancy of 11 people. In 50% of the homes, the mother was the primary caretaker of the child during the day. PHQ-9 scores at screening averaged 12.13 (SD 2.85) with BDI and WHODAS scores on day one having a mean of 25.63 (SD 5.45) and 14.08 (SD 9.17) respectively. By the end of the study interview over six weeks later these scores had dropped to 5.48 (SD 4.65) and 8.81 (SD 8.56) respectively.

**Table 1:** Demographic characteristics of the sample.

	Depressed mothers
	n (%)/Mean (SD)
Ethnicity	
Dalit	10 (41.67%)
Darai	4 (16.67%)
Tharu	2 (8.33%)
Brahman/Chhetri	2 (8.33%)
Magar	2 (8.33%)
Other	4 (16.67%)
Religion	
Hindu	20 (83.33%)
Christian	2 (8.33%)
Other (Parmesowar pita; Biswasi pita permswor)	2 (8.33%)
Index child Age (months)	5.75 (3.60)
Child Sex	
Male	10 (41.67%)
Female	14 (58.33%)
Mother’s Age	21 (2.06)
Mother’s education	
Primary	5 (20.83%)
Secondary	17 (70.83%)
Higher Secondary	2 (8.33%)
Mother’s occupation	
Housewife	17 (70.83%)
Agriculture	2 (8.33%)
Unemployed	2 (8.33%)
Daily ware labour	2 (8.33%)
Business	1 (4.17%)
Years Married	4.46 (3.08)
Number of children (median, min–max)	2 (1–3)
Age of oldest child	6.06 (5.10)
Age at first pregnancy	17.54 (1.79)
Index child’s caretaker during the day	
Self	12 (50.00%)
Sister	3 (12.5%)
Other family	9 (37.5%)
Fathers education	
Primary	5 (20.83%)
Secondary	17 (70.83%)
Higher secondary	2 (8.33%)
Fathers occupation	
Office	10 (41.67%)
Daily wage labour	8 (33.33%)
Working abroad	3 (12.50%)
Unemployed	2 (8.33%)
Driving	1 (4.17%)
Internet at home (count, percent)	0 (0%)
Electricity at home	
Yes	23 (95.83%)
No	1 (4.17%)
Number of household occupants	5.66 (2.28)
Household occupants < 18 years (median, min, max)	5 (3–11)
Mental health	
PHQ-9 at screening	12.13 (2.85)
BDI Day 1*	25.63 (5.45)
BDI Week 2	22.79 (7.55)
BDI Week 3	16.65 (6.98)
BDI Week 4	13.22 (8.95)
BDI Week 5	11.13 (11.08)
BDI Week 6	6.46 (5.36)
BDI end of study	5.48 (4.65)
WHODAS Day 1*	14.08 (9.17)
WHODAS end of the study	8.81 (8.56)

*First day of passive sensing data collection.

**Figure 2:**
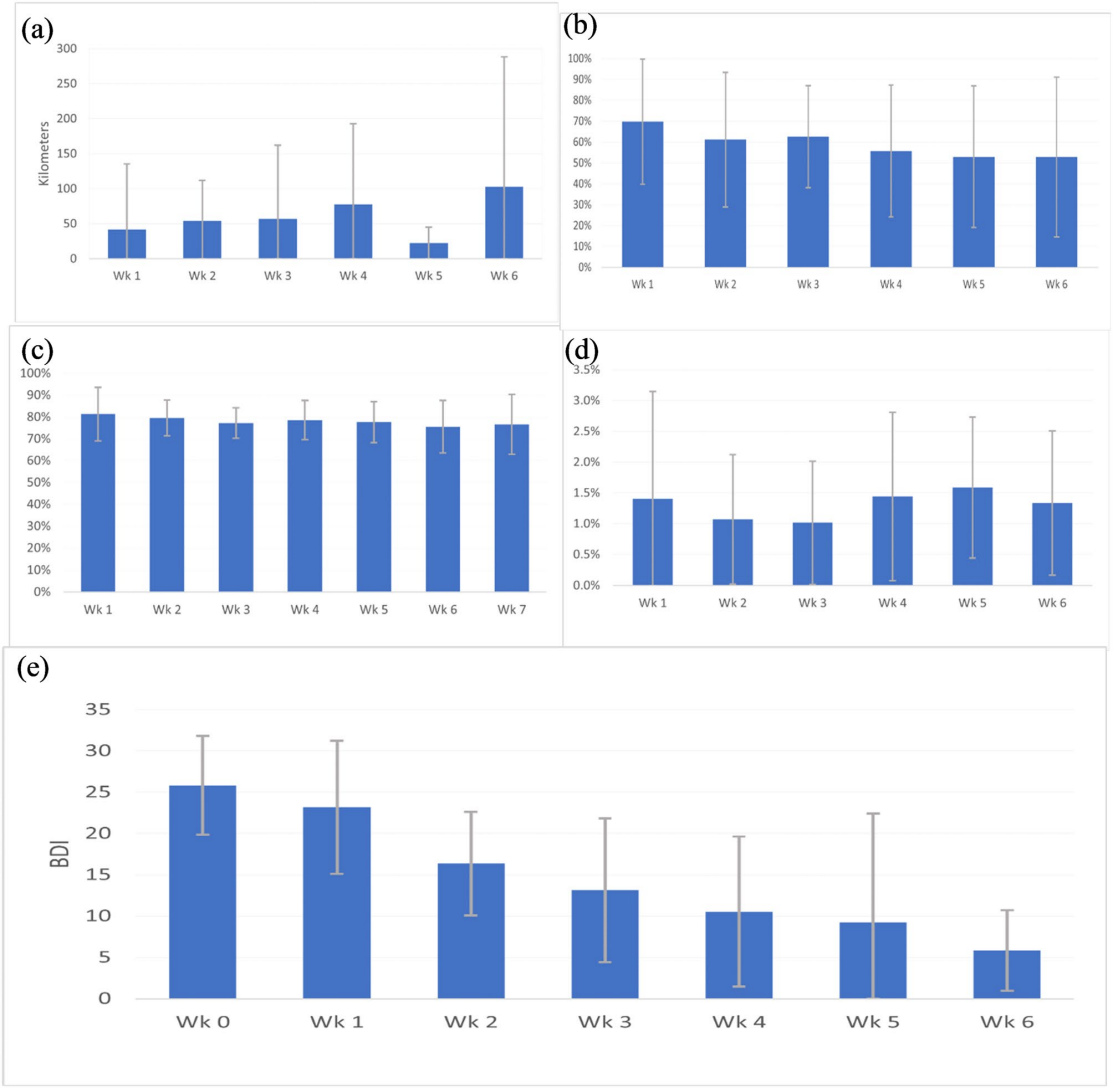
Depression, and sensing data by week with confidence intervals; (**a**) average straight-line distance travelled per week; (**b**) the percentage of time mother and child were detected in (10 m) proximity of each other; (**c**) percent of weekly recordings in which speech was detected; (**d**) percent weekly walking activity detected; and e) BDI score by week (week 0 = at screening).

Figure 2 provides an overview of weekly data patterns seen in data from the four passive sensors. The sum of the average straight-line distance travelled increases from 41.9 to 103 km over the course of the six weeks. Time spent in proximity (10 m) between mother and child (beacon and phone) is seen to decrease from 70% in week 1 through to 53% in week 6. The amount of speech detected and the percentage of time walking remain fairly consistent across the six weeks. BDI scores are presented for comparison and can be seen to fall from 25.6 at screening to 5.8 at the final measurement.

**Figure 3:**
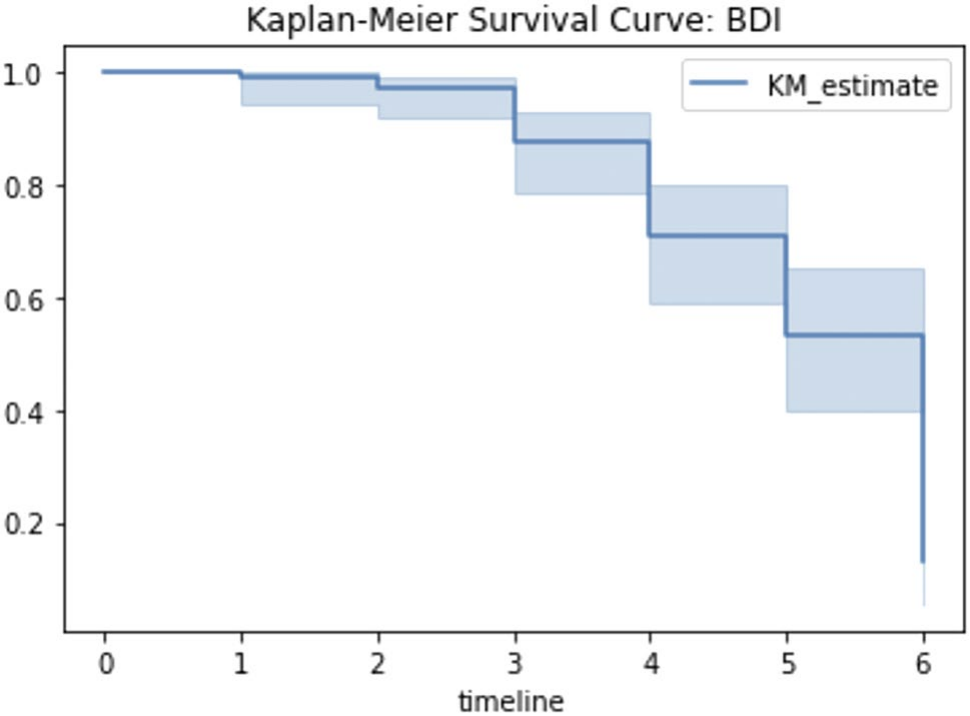
Kaplan Meyer survival curve for BDI score and weeks of treatment.

Pearson correlations for these data are presented in Table 2 with a moderate negative correlation between week of intervention and BDI scores. The percentage of time together was weakly negatively correlated with intervention week. Figure 3 presents the survival curve for BDI score through time. Although most mothers remained above the BDI cut-off of 10 over the first three weeks, by week 5, 53% of mothers were no longer above the cut-off for mild depression.

**Table 2:** Spearman rho correlation for week, BDI, and passive sensors.

	Week	BDI	Distance straight line	%Together	%Foot	%Speech
Week	1.00	− 0.46*	0.07	− 0.25	− 0.11	− 0.08
BDI	− 0.46*	1.00	− 0.16	− 0.18	0.15	0.03
Distance straight line	0.07	− 0.16	1.00	− 0.08	− 0.14	0.19
%Together	− 0.25	− 0.18	− 0.08	1.00	0.22	0.19
%Foot	− 0.11	0.14	− 0.14	0.22	1.00	0.18
%Speech	− 0.08	0.03	0.19	0.19	0.18	1.00

*p < 0.01 after Bonferroni correction for multiple comparisons.

The only statistically significant change registered between week 1 and week 6 was the BDI score (Table 3). Walking activity, time in close proximity, and presence of speech were not associated with significant change between weeks 1 and 6. GPS data was not available in week 1 and week 6 for 23 of the participants making it infeasible to look at change through time. Qualitative insights into participants experience of passive sensing being integrated into counselling sessions is provided in Table 4.

**Table 3:** Changes from Week 1 to Week 6 for passive sensors data and mental health outcomes of interest.

	Mean wk1—wk6 difference	SD	95% Confidence Interval Difference		t	df	Sig, (2-tailed)
			Lower	Upper			
BDI	− 19.79	6.95	− 23.01	− 16.99	− 13.76	23	< 0.01
Straight line distance (km)	1.35	–^&^					
Walking activity (%)	− 0.14	2.45	− 1.12	1.11	− 0.24	18	0.81
Time in close proximity (%)	− 17.43	41.37	− 37.00	3.00	− 1.84	18	0.08
Time where speech detected (%)	− 3.39	13.61	− 10.0	3.00	− 1.14	20	0.27

^&^Only one participant and both a week 1 and week 6 straight line distance travelled measurement making further difference analysis impossible.

**Table 4:** Qualitative insights from mothers enrolled in the Standstrong study.

On Integrating BDI, PHQ9, scores in the StandStrong App for mothers to monitor their mood
** Interviewer**: Can you remember about the counselling session from the start to the end? What type of problems did you have in the beginning and how do you feel now? ** Participant**: Previously, before using these technologies, I was worried, when I met her on the first session**,** my [BDI] score was 35, but in the second session it becomes 10. It was decreasing 25-year-old mother with a 6-month old infant
Empowerment and improved compliance with HAP recommendations
** Interviewer**: What did she [counselor] identify after checking [the app] and how did you feel after that? ** Participant**: She identified that my problems were decreasing ** Interviewer**: Then, what did she show in that Tab[let]? ** Participant**: Information about my movement, distance between mother and baby etc ** Interviewer**: What did you feel when she show those information? ** Participant**: I felt happy. Because at that time, I came to know that it records all the places where I move, what I did while using this mobile ** Interviewer**: Among the information, which one do you like mostly? ** Participant**: I liked the information about my movement, I felt happy when I read the name of places where I have gone. I was motivated to walk here and there 25-year-old with a 6-month old infant
Improved compliance with technology use
In the first week, she [the participant] didn’t carry that mobile, forgot to carry that mobile. But after taking HAP session, and after^24^ showed mobile data in the HAP session, she [the participant] came to know about the distance between mother and baby, her movement etc. and get motivated to carry that mobile and put that beacon to her baby Research Assistant fieldnotes from interview with a 20-year-old mother of a 4-month old infant
Counsellor on the use of app during therapy session
** Interviewer**: In your opinion, what kind of differences did you find using app and without using app? Just like in HAPs session, you conducted sessions without the app and when we were in component 3 we totally used sensing data. What differences did you find? ** Counsellor**: First it was the timing. It took more time when we added app. Another one was we could show them the data according to the [behavioural] changes^31^ the [sessions] where we did not use the app, we trusted^18^ they said. That was the difference….To encourage the participants, it was easier to show them [the app] which was the additional benefit

## Discussion

A five-session HAP intervention delivered by trained lay counsellors in Nepal showed a significant reduction in symptoms of depression. These findings add to the growing body of literature on the effectiveness of task-shifting mental health support to lay counsellors trained in interventions that utilise the principles of BA^36^. Noticeable trends in passive sensing data were observed across the 6 weeks of intervention. GPS data is one of the more well-established passive sensing data types with, for example, studies showing increased homestay among depressed adults^37^. Data from this study support these findings with the weekly average of straight-line distance travelled increasing in a somewhat linear pattern. The small sample size does mean this trend needs to be interpreted with caution as measures of dispersion show large weekly variability. Proximity between mother and infant is novel and not well explored in the literature. In examining Bluetooth proximity data, it is important to consider the role of the mother in child caregiving. Preliminary analyses suggest that mothers identified as primary caretakers are more frequently within a 10-m range of their children. Further statistical analysis could elucidate whether these differences are significant, providing deeper insights into maternal engagement patterns based on their caregiving role. Additionally, the 10-m threshold used in this study typically encompasses the indoor space (affected quite heavily by furniture and the thickness of the walls) of a standard home in the study area. Therefore, mothers might still be within this range while performing indoor or be outside performing household tasks but be picked up as “apart” by our Bluetooth system. Future studies might benefit from a finer resolution of proximity measurements or the incorporation of environmental context to better capture the dynamics of mother–child interactions. Alternatives to Bluetooth such as Ultrawideband radios exist and provide resolutions closer than the 10 m accuracy typical of Bluetooth-based systems. Our data show a slow decrease in the amount of time spent within the general proximity of each other. This interpretation is supported by the extended max distance travelled as the weeks went on. On the other hand, it is not possible to refute the possibility that the beacon was just being worn less by the child as research fatigue set in. If the beacon was turned off more frequently or was not put into the pouch given to each child to carry the beacon, we might anticipate a similar pattern.

Audio data show consistently high levels of speech detection throughout the six weeks. An initial analysis of these data using the AudioSet model, trained and released by Google, found that 43% of all samples contained speech data^18^. For this paper, audio data were passed through the pyannote voice activity detection model. Although less capable of detecting the range of environmental audio that the AudioSet model can handle, the pyannote model sacrifices breadth for narrow speech detection accuracy. The model is lightweight and could be deployed on an edge device such as a mobile phone or watch as one way to increase the acceptability of audio recordings in the home. The detection of speech in 80% of the audio samples raises important considerations regarding the auditory environment of the home. It is evident that deeper insight could be gained if it was possible to distinguish between maternal speech directly engaged with the child and other household speech captured by the device such as from the television, radio, or other adults in the house talking to each other. This distinction is fundamental, as shown by^38^ infants who experienced more child-directed speech became more efficient in processing familiar words in real time and had larger expressive vocabularies by the age of 24 months, although speech simply overheard by the child was unrelated to vocabulary outcomes. Future analyses could include categorizing the types of speech interactions and their potential developmental impacts on the child.

A key principle of BA, on which HAP is built, is that movement (physical activity) can be supportive of improved mood. However, we did not observe an increase in physical activity (i.e., walking). Most data recorded a state of “still” (i.e., not moving). A number of possible explanations exist. One interpretation might be that the mothers were actually not very active and that they maintained this low rate of activity throughout the study. The second possible interpretation is that the granularity of the recoding was too coarse to pick up the walking activity that took place in the gaps between the 15-min recoding interval. Finally, it might be that the mobile phone was simply not always carried on the mother’s person and rather was placed on a table or other surface in the home. Together, these data are interesting but cannot be generalized given the small sample size and the heterogeneity among the mothers’ patterns throughout HAP treatment.

The limitations uncovered during the implementation of digital phenotyping in the home of mothers with PPD are interesting and possibly useful to others who would undertake similar work. First, as reported by^20^ data volumes in our study are seen to decrease the longer data are collected. Increased effort is needed from the field team as the implementation timeframe grows longer to ensure important data are not lost. Lay counsellors can also potentially promote the continued use of devices by maintaining the use of passive data findings throughout HAP delivery. Validity checks are also highly recommended. These could be as simple as a randomly timed check-in every few days to enquire about where they are, what they are doing, who they are with, and whether they have the app and beacon attached and on. These data can then later be used to corroborate what is seen in the passive data.

Another possible approach is to move the passive data collection from the phone to the smart watch. The watch is easier to carry around all day at home than a phone and additionally, the collection of heart rate data, available on almost all smartwatches these days, could be used to filter out other sensor data collected when a heartbeat is not present at that time point. In other words, if the watch is been worn, a heartbeat will be detected. This improves the interpretability of the data is it removes the concern that the watch may be lying on the bedside table. One final approach that may improve data interpretability is the triangulation of different data streams into a single multimodal analysis. For instance, if the proximity sensor suggests that the mother is not with the child, it may be difficult to interpret what exactly is triggering this reading. If the proximity data is triangulated with activity, and it is noted that the mother is walking and GPS data suggests she is outside the house, then it is more plausible to infer that she is indeed away from her child.

Once solutions similar to those suggested above have been implemented and interpretation and data validity are more solid, the possibility becomes real that person-centred interventions could be developed around the different latent class sub-types presented in the data. Although criticism exists that digital phenotyping depersonalises care by bucketing people together rather than treating each person as an individual^39,40^, the reality is that many lay counsellor-led interventions end up being reduced to fixed steps from a manualised intervention with limited variety and personalisation being provided^41^. With the approach outlined in this paper, the opportunity exists to support lay counsellors to provide high-quality care that is responsive to the individual life patterns their clients are experiencing. In addition to the effort needed to ensure that the passive data are reliable and interpretable, counsellors also require significant training if they are to understand how these data can be integrated into the care they provide. Simply providing a dashboard of data will not suffice. Training, examples, and guidelines are all needed to help lay counsellors understand what the passive data means for the therapy they are providing to each individual client.

Passive sensing technology, to date, has predominantly been explored in the area of detection and diagnosis of mental health conditions. However, there is tremendous potential for passive sensing to enhance the delivery of psychological interventions. This is the first application, to our knowledge, of paraprofessional counsellors in a low-resource setting who are guiding their treatment of postpartum depression among adolescent and young mothers using passive sensing technology. Passive sensing can be impactful in delivering personalized care because of real-time measurement of behaviour, including mother-infant interaction, which is important to improve both mothers’ and infants’ health outcomes.

## Data Availability

Dataset generated and or analysed during the current study are available from the corresponding author upon request.
